# The Enterovirus 71 A-particle Forms a Gateway to Allow Genome Release: A CryoEM Study of Picornavirus Uncoating

**DOI:** 10.1371/journal.ppat.1003240

**Published:** 2013-03-21

**Authors:** Kristin L. Shingler, Jennifer L. Yoder, Michael S. Carnegie, Robert E. Ashley, Alexander M. Makhov, James F. Conway, Susan Hafenstein

**Affiliations:** 1 Department of Medicine, Department of Microbiology and Immunology, The Pennsylvania State University College of Medicine, Hershey, Pennsylvania, United States of America; 2 Department of Structural Biology, University of Pittsburgh School of Medicine, Pittsburgh, Pennsylvania, United States of America; Institut Pasteur, France

## Abstract

Since its discovery in 1969, enterovirus 71 (EV71) has emerged as a serious worldwide health threat. This human pathogen of the picornavirus family causes hand, foot, and mouth disease, and also has the capacity to invade the central nervous system to cause severe disease and death. Upon binding to a host receptor on the cell surface, the virus begins a two-step uncoating process, first forming an expanded, altered “A-particle”, which is primed for genome release. In a second step after endocytosis, an unknown trigger leads to RNA expulsion, generating an intact, empty capsid. Cryo-electron microscopy reconstructions of these two capsid states provide insight into the mechanics of genome release. The EV71 A-particle capsid interacts with the genome near the icosahedral two-fold axis of symmetry, which opens to the external environment via a channel ∼10 Å in diameter that is lined with patches of negatively charged residues. After the EV71 genome has been released, the two-fold channel shrinks, though the overall capsid dimensions are conserved. These structural characteristics identify the two-fold channel as the site where a gateway forms and regulates the process of genome release.

## Author Summary

In a picornavirus capsid structural integrity must not be compromised until a key mechanism triggers genome release into a permissive cell. It has long been established that the majority of members of the picornavirus family solve this dilemma with a two-step uncoating process initiated by receptor recognition. For human enteroviruses, binding of an entry receptor triggers a series of conformational changes, resulting in an “A-particle” that is primed for genome release. After endocytosis, an unknown trigger causes the A-particle to expel the viral genome, leaving behind an emptied capsid. This process can be mimicked in solution by heating mature virus. Though the capsid species for both of these steps have been isolated, the fine details of the uncoating process have yet to be elucidated. Cryo-electron microscopy reconstructions of the enterovirus 71 A-particle and empty capsid provide compelling structural evidence to suggest that the icosahedral two-fold axis opens a channel that acts as a gateway in the viral capsid, regulating the release of genomic material from the altered particle.

## Introduction

Enterovirus 71 (EV71) is an emerging pathogen with pandemic potential [1]. First isolated in 1969, EV71 is classified as a member of the family *Picornaviridae*, genus *Enterovirus* [2], [3]. This single-stranded positive-sense RNA virus is a causative agent of hand, foot, and mouth disease, a childhood illness that is usually mild and self-limiting. However, for some infected individuals, EV71 invades the central nervous system causing severe disease ranging from meningitis to fatal encephalitis [4]. Recently, outbreaks of EV71 have been common in the Asia-Pacific region, with high prevalence of severe disease and mortality being reported [5]. The link between EV71 and worldwide health risks has spurred increased research, as there is no effective treatment or vaccine.

**Figure 1:**
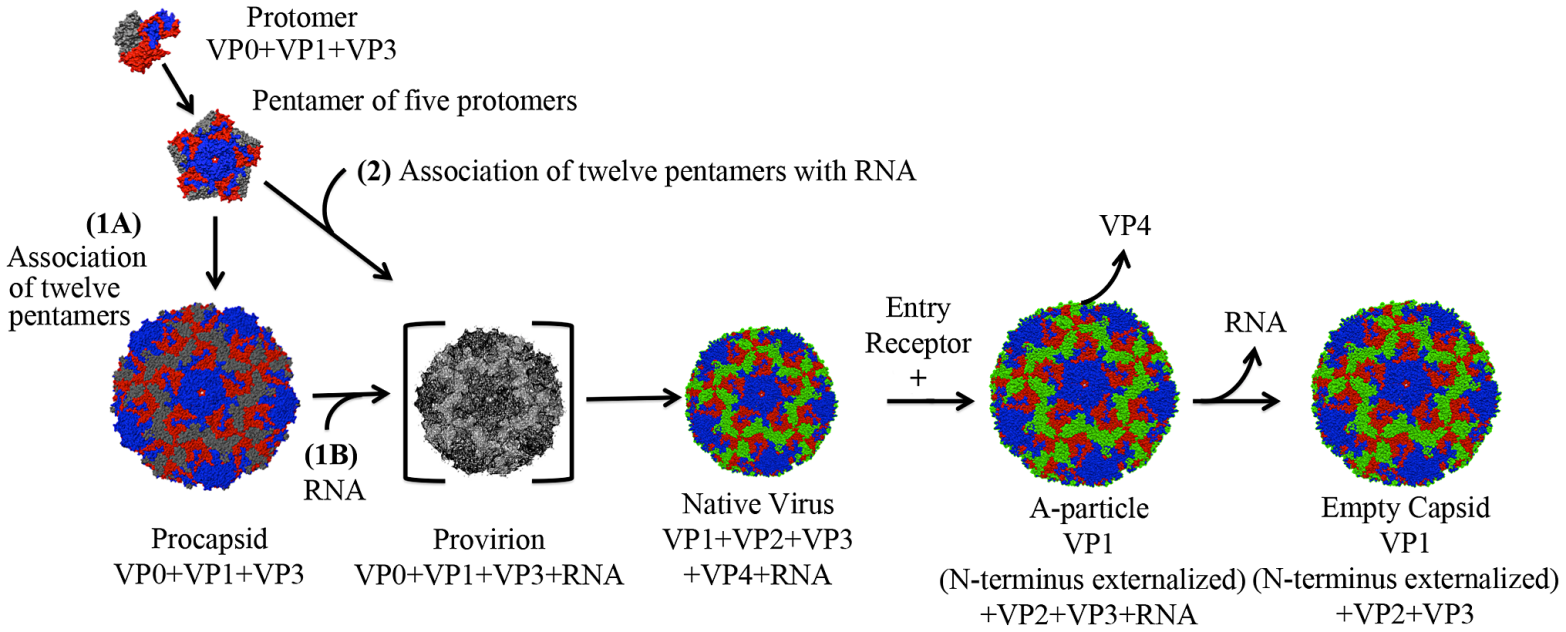
Lifecycle of a human enterovirus. Three viral structural proteins (VP0, VP1, and VP3) function as a single structural subunit, the protomer. Five protomers then form a pentamer, twelve of which can self-associate to form a naturally occurring empty capsid (often referred to as procapsid). In pathway one, after the twelve pentamers form an empty procapsid (1A) the genome is inserted (1B). In the second proposed pathway 12 pentamers assemble around a genome to form a provirion (2). In this pathway pentamers that associate in the absence of genome would serve as a reservoir of capsid components in infected cells, and procapsid would be an off-pathway assembly byproduct. The provirion is a short-lived intermediate, as the autocatalytic cleavage of VP0 to form VP2 and VP4 is rapid and efficient. This cleavage results in the native virus, which upon binding to a cellular receptor transitions to an A-particle, which has expelled VP4 and exposed the N-terminus of VP1. An unknown secondary trigger causes the genome to egress, leaving behind an empty capsid shell. Note: The procapsid in this figure is shown in an expanded form, like that of EV71. Other picornavirus procapsids are the same diameter as their native virion (poliovirus and foot and mouth disease virus [20], [21]).

Extensive structural and biochemical studies have identified five distinct particles that represent the lifecycle of a human enterovirus: putative procapsid, provirion, mature infectious virus, the altered particle termed the “A-particle”, and empty capsid (Fig. 1) [6], [7], [8], [9], [10], [11], [12]. Assembly begins in the cytoplasm where translation of the viral structural polyprotein and subsequent cleavage allows the formation of protomers consisting of VP0, VP1, and VP3. Five protomers then join together to form the pentameric assembly subunit, twelve of which self-assemble into a naturally occurring empty capsid (procapsid) [13], [14], [15]. Two distinct assembly pathways have been proposed. In one pathway, this procapsid is a true intermediate, into which the genome would be packaged by some yet unknown mechanism to form provirion [16], [17], [18]. Alternatively, in a second proposed pathway, twelve pentamers associate around a viral genome, directly forming a provirion [19]. In this case, the procapsid would serve as a reservoir of capsid components, and represent an off-pathway byproduct of assembly. Regardless of their role in assembly, procapsids are found in all infected cells, and contain 60 copies each of the structural proteins VP1, VP3, and VP0, but are devoid of genome [20], [21], [22]. After the provirion is formed, interactions between the structural proteins and the RNA genome cause the autocatalytic cleavage of VP0 into VP2 and VP4 [15], [23], [24]. Whereas portions of VP2 map to the capsid exterior, all of VP4 is internal [6], [7]. The resultant mature virus particles are released from infected cells to continue the lifecycle.

Mature viruses must interact with a receptor on the surface of a host cell to initiate the uncoating process and successfully infect the host. For many picornaviruses these interactions rely on the highly conserved architecture of the human enterovirus capsid and the use of a cellular receptor that is composed of an immunoglobulin-like (Ig-like) domain [25], [26]. Surrounding each five-fold axis is a depression, forming a narrow channel referred to as the canyon [6], [7]. A small opening is present at the bottom of the canyon, which leads to a hydrophobic “pocket” filled with a lipid moiety named the “pocket factor”. The presence of the pocket factor likely stabilizes the mature capsid [27]. One current model for picornavirus uncoating states that the Ig-like receptor binds into the canyon, dislodges the pocket factor, and allows the proteins to shift as rigid bodies. These events lead to the formation of the expanded A-particle. During the expansion process the N-terminus of VP1 is externalized, and VP4 is expelled from the capsid [28], [29]. These alterations poise the particle for genome release. Subsequently, a second and unknown trigger causes the genome to egress, leaving behind an empty capsid shell [28]. The resulting productive infection will allow the capsid assembly and morphogenesis process to continue anew within the next host.

Recent x-ray crystallography studies have revealed the structures of the EV71 procapsid and mature virus [30], [31]. The procapsid is ∼4% larger than the native virus and has a large hole present at each two-fold axis of symmetry [30], [31]. The canyon is fully formed, but is shallow compared to other known human enterovirus structures. Additionally, the pocket is collapsed, making it inaccessible to lipids. Relative to the procapsid, the structural protein shell of the mature virus is condensed, and each protomer is rotated 5.4° clockwise at each three-fold axis [30], [31]. These protein movements open the pocket, allowing for a lipid to be inserted. The EV71 pocket factor was found to be longer compared to those of other picornaviruses, and is exposed at the tip [31]. Additionally, the holes at each two-fold axis are closed. These structural rearrangements place the mature virus into a conformation capable of proceeding through the uncoating process.

Five cellular receptors have been identified for EV71, P-selectin glycoprotein ligand-1 (PSGL-1), annexin II, heparin sulfate, sialyted glycoprotein, and scavenger receptor B2 (SCARB2) [32], [33], [34], [35], [36], [37]. The exact function of each of these proteins in virus attachment and entry has yet to be discerned. Surprisingly, none of the identified receptors have an Ig-like domain. However, the shallow nature of the EV71 canyon suggests that an immunoglobulin fold may not be necessary to penetrate the canyon, displace the pocket factor, and trigger the uncoating process [30], [31]. Though the binding footprint for SCARB2 is not known, mutational evidence suggests that it may act in this manner to trigger A-particle formation and subsequent genome release [38]. Importantly, receptor use is not the only way to initiate picornavirus uncoating. Many studies have shown that heating the virus *in vitro* is an acceptable surrogate for receptor binding [39]. Wang *et al.* has reported that heated EV71 mature capsids crystallize isomorphously with procapsid, indicating that both types of empty particles are expanded and retain the same surface features [30].

To elucidate the structural rearrangements that occur during the EV71 uncoating process, we have reconstructed high-resolution cryo-electron microscopy (cryoEM) maps of the A-particle (6.3 Å) and empty capsid (9.2 Å). Structurally similar to the procapsid [30], [31], these new cryoEM structures have an expanded form with large open channels at the icosahedral two-fold axes of symmetry that are lined with patches of negatively charged residues. The A-particle two-fold openings are larger in diameter and the internal capsid residues link the protein shell to the viral RNA. The location of the N-terminal portion of VP1 that is disordered in the procapsid crystal structure, maps to pentameric pillars of density that connect to the base of the canyon. The A-particle and empty capsid have remarkably similar structures, with small differences in protein composition. The cryoEM maps of the A-particle and empty capsid provide a detailed view of uncoating for EV71. These structural characteristics specifically identify the channel at the two-fold axis as the site for the formation of a gateway, through which the process of viral genome release is regulated.

## Results

### CryoEM reconstructions

**Figure 2:**
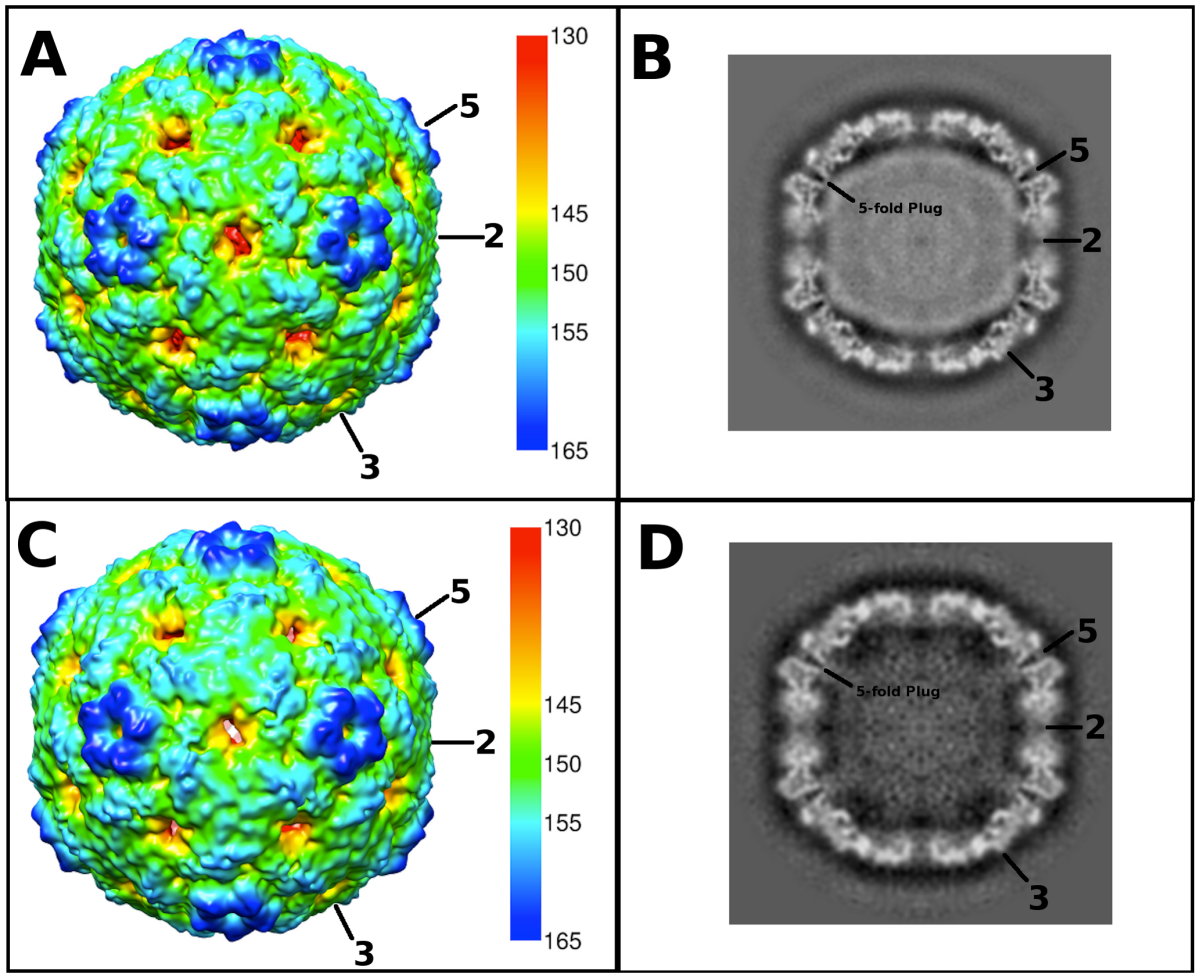
Cryo-EM reconstructions of the EV71 A-particle and empty capsid. (A, C) Surface rendered cryo-EM maps of the 6.3 Å EV71 A-particle and the 9.2 Å EV71 empty capsid are shown at a contour level of 1σ colored radially as indicated by the scale bar that depicts the most internal buried density in red gradating to the most external density in dark blue). The five-fold, three-fold, and two-fold axes are labeled. (B, D) Central sections of the EV71 A-particle and empty capsid with density shown in grey. The A-particle contains density corresponding to viral RNA (grey interior), whereas the empty capsid is completely devoid of interior density, indicating the genome has been released. Symmetry axes are labeled and the plug of density that closes the pore through the five-fold vertex is indicated on the central sections of the A-particle and empty capsid.

Purified EV71 mature virus was heated to 56°C for 12 minutes to produce A-particles (see Materials and Methods). Electron microscopy revealed that the heated capsids were in one of two forms: 1) the A-particle, with genetic material remaining encapsidated (∼85%), or 2) the empty capsid containing no genetic material (∼15%) (Fig. S1). As A-particles could be easily distinguished from empty capsids, the sample was vitrified and used for cryoEM data collection. These data were used to produce 3D reconstructions of the A-particle (6.3 Å) and the empty capsid (9.2 Å) (Table 1, Fig. 2A, C). Both capsid forms were radially expanded compared to the native virus and measured ∼30 nm in diameter [30], [31]. The A-particle interior contained density corresponding to the RNA genome, whereas the empty capsid was completely devoid of internal density (Fig. 2B, D). Both capsid conformations had open channels at the two-fold axis of symmetry, consistent with the expanded native empty capsid crystal structure [30]. The five-fold vertex opened at the surface of the capsid in both structures, though the resulting channel through the capsid shell was plugged in the interior. The canyon existed as a thin shallow depression around each five-fold vertex.

**Table 1:** Cryo-electron microscopy image reconstruction data.

Structure	Films	Defocus range (µm)	Particles total	Particles used	Resolution (Å)
A-Particle 1	40	1.73–4.28	14,295	11,436	6.3
A-Particle 2	5	1.89–3.87	2,334	2,334	8.7
Empty Capsid	40	1.73–4.37	2,146	1,931	9.2

### Protein from the interior capsid of the A-particle extends into the RNA density

**Figure 3:**
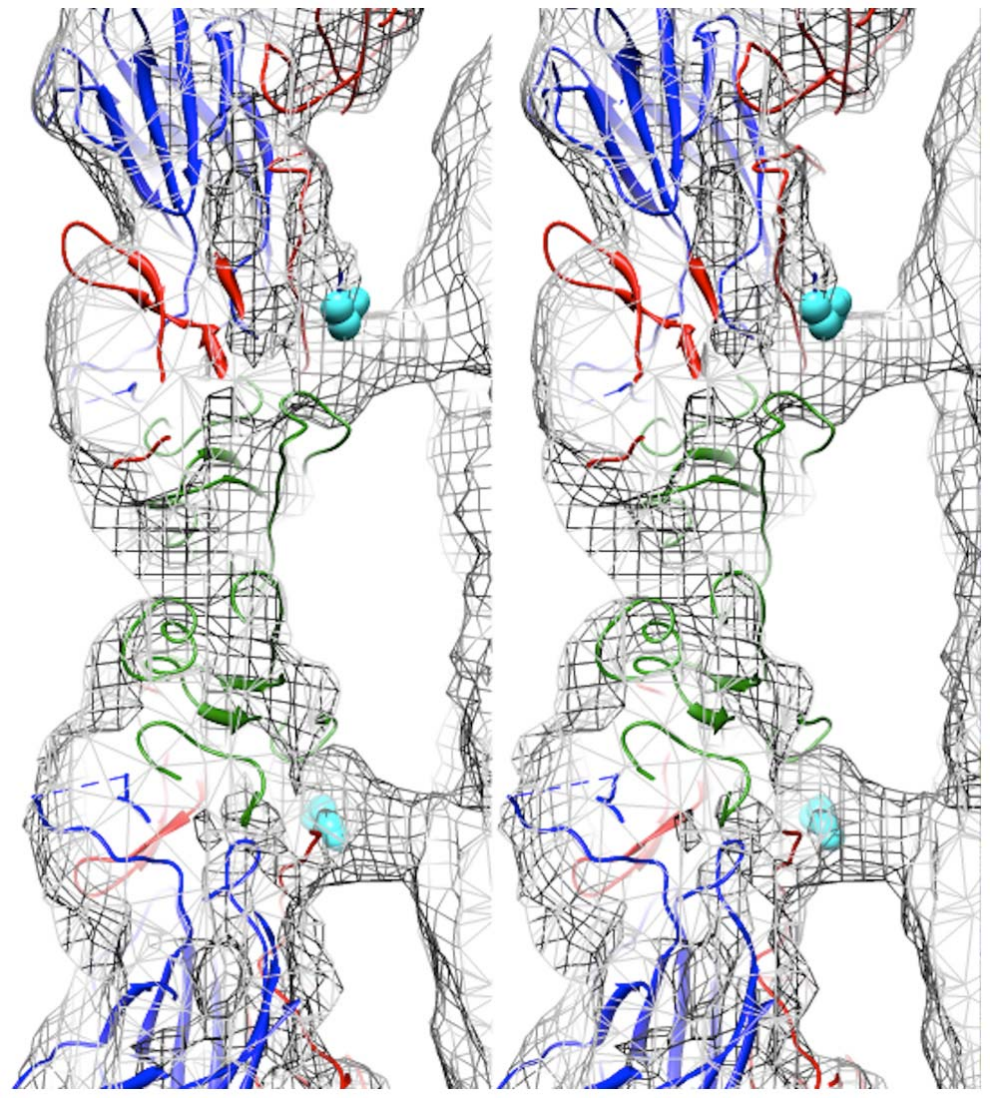
Capsid-RNA interactions in the EV71 A-particle. Stereogram of the EV71 A-particle showing two columns of protein density extending from the capsid shell to interact with the viral RNA genome. The fitted procapsid crystal structure (3VBU) is depicted as a ribbon diagram with VP0, VP1, and VP3 in green, blue, and red, respectively [30]. The A-particle cryoEM density is depicted as grey mesh. The first ordered N-terminal residue of VP1 in the crystal structure, His73, is shown as cyan spheres and extends into the protein density. The unfilled density beneath His 73 is attributed to the N-terminus of VP1, which is disordered and therefore absent in the procapsid crystal structure, but present in the A-particle.

Fitting of the procapsid crystal structure (PDB accession code 3VBU) into the EV71 A-particle cryoEM density map (cc = 0.82) identified specific protein-RNA interactions [30]. The first ordered residue in the N-terminal portion of VP1 in the procapsid crystal structure is His73. This residue is positioned directly within the density pillar that connects the capsid shell to the interior RNA (Fig. 3). Without further analysis, the unfilled cryoEM density in the EV71 A-particle map could be attributed to RNA, the N-terminus of VP1, or a combination of the two molecules.

### The N-terminus of VP1 is externalized at the base of the canyon

**Figure 4:**
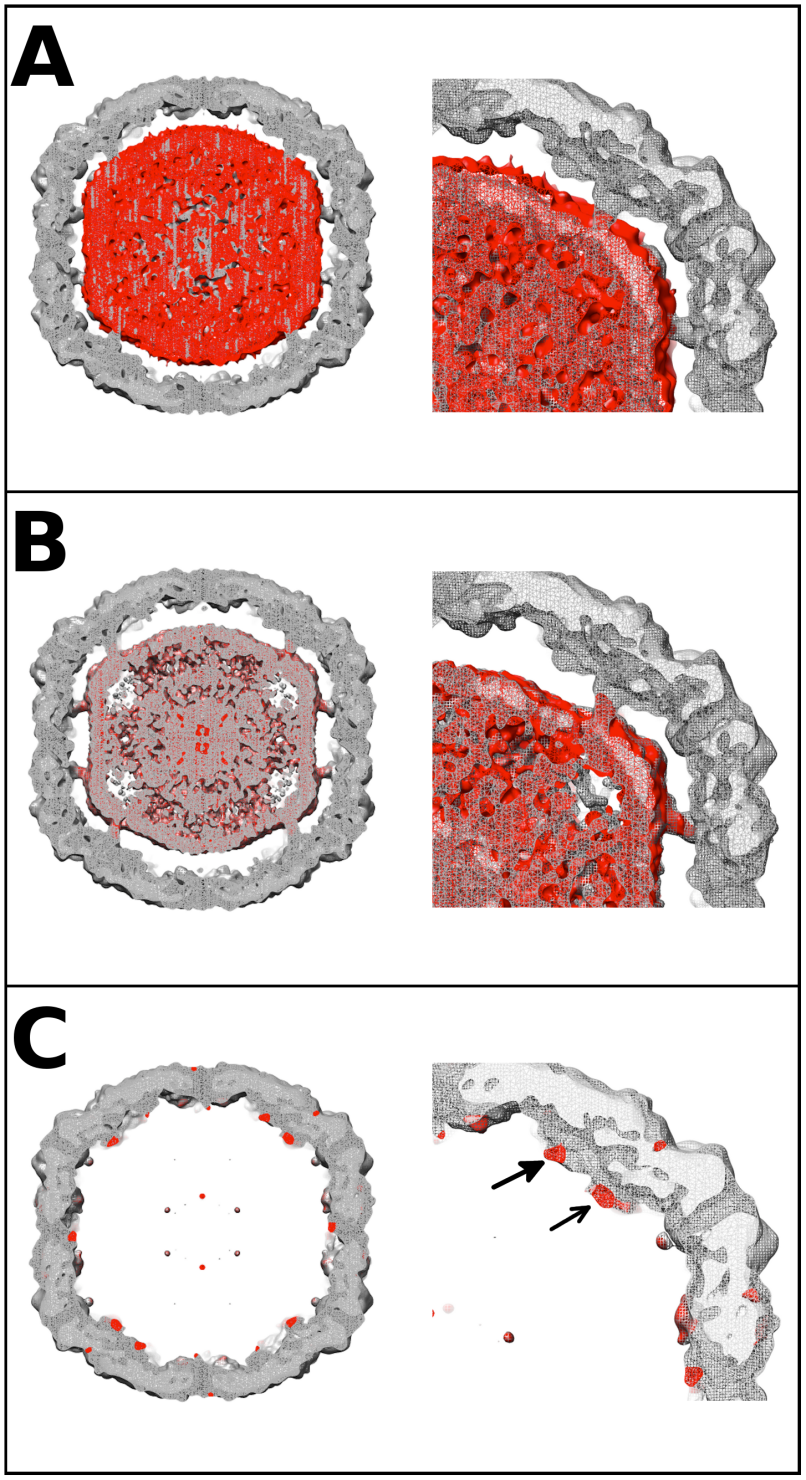
EV71 difference map interpretation. Central sections of calculated difference density maps and close-ups of relevant features are shown to indicate the position of the N-terminus of VP1. (A) The empty capsid density map was subtracted from 9 Å A-particle density map. The difference density (red) is shown superimposed on the A-particle map. The difference density corresponds to the RNA in the particle center, and does not fill the pillars that extend inward from the capsid shell. This can be seen clearly in the close-up of the right upper quadrant of the central section. (B) The calculated procapsid density map was subtracted from the 6.3 Å A-particle density map. The difference density (red) is shown in relation to the A-particle map (grey). The difference density fills the pillars that surround the 5-fold axis and also corresponds to the viral genome in the interior of the particle. The close-up of the upper right quadrant shows that the pillar is mostly filled with difference density (red). (C) The calculated procapsid map was subtracted from the empty capsid density map. The resulting difference density (red) is shown on the empty capsid map (grey). The close-up view shows that the difference density is localized to the pillar (solid arrow head), base of the canyon (unfilled arrow head), and capsid exterior near the 5-fold axis. These same densities are seen in their symmetry related positions with a small noisy density at the tip of the five-fold plug also present.

Difference map analysis was used to identify the nature of the unfilled density remaining after fitting the procapsid crystal structure into the cryoEM map of the EV71 A-particle. In order to ensure this analysis was highly rigorous, equal quality maps were generated for the EV71 procapsid structure, and the EV71 A-particle map. Three difference density maps were calculated to make the analysis: A-particle minus empty capsid, A-particle minus procapsid, and empty capsid minus procapsid (Fig. 4). The density differences between the EV71 A-particle and empty capsid were limited to a region in the capsid interior that did not contact the protein shell (Fig. 4A). The lack of interaction between the capsid interior and difference density unequivocally indicates that it corresponds to viral RNA. Subtracting the density of the procapsid from the A-particle left density in the center of the particle that protrudes outward in columns towards a region between the two-fold and five-fold axes (Fig. 4B). This density does contact the interior of the capsid shell, indicating that it can be attributable to both protein and RNA. The density that remained when the procapsid was subtracted from the empty capsid corresponded to a region at the base of the canyon on the capsid exterior and as extra density at the interior shell directly below the canyon. There is an additional difference density on the interior of the empty capsid that is in the same position as the density pillars in the A-particles (Fig. 4C). The procapsid and empty capsid are both void of genome, thus this density must be ascribed to protein. Therefore the portion of the pillar density present as difference density in Fig. 4C and the canyon-associated density must be attributed to protein that is present in the A-particle and empty capsid, but missing or disordered in the procapsid crystal structure. Neither the A-particle nor empty capsid contains VP4, so the only protein that could fill these densities is the first 72 N-terminal residues of VP1.

### Volume measurement of the N-terminus of VP1

To determine if the disordered residues of VP1 in the procapsid crystal structure could fill the density pillar and extend through the capsid shell, a density volume analysis was done. Icosahedral symmetry operators were applied to amino acid residues 1–72 of VP1 from the crystal structure of mature virus (3VBS) [30] and surface contoured at 1 sigma. The volume of this structure was measured to be 116.8e^3^ Å^3^ (data not shown). The volume of the empty capsid minus procapsid difference map when displayed at a contour level of 1 sigma was 97.81e^3^ Å^3^ (data not shown). Thus, the disordered N-terminus of VP1 in the EV71 procapsid, is large enough to occupy a portion of the pillar density, extend through the capsid, and become externalized in the A-particle and empty capsid.

### Two-fold symmetry axis channel

**Figure 5:**
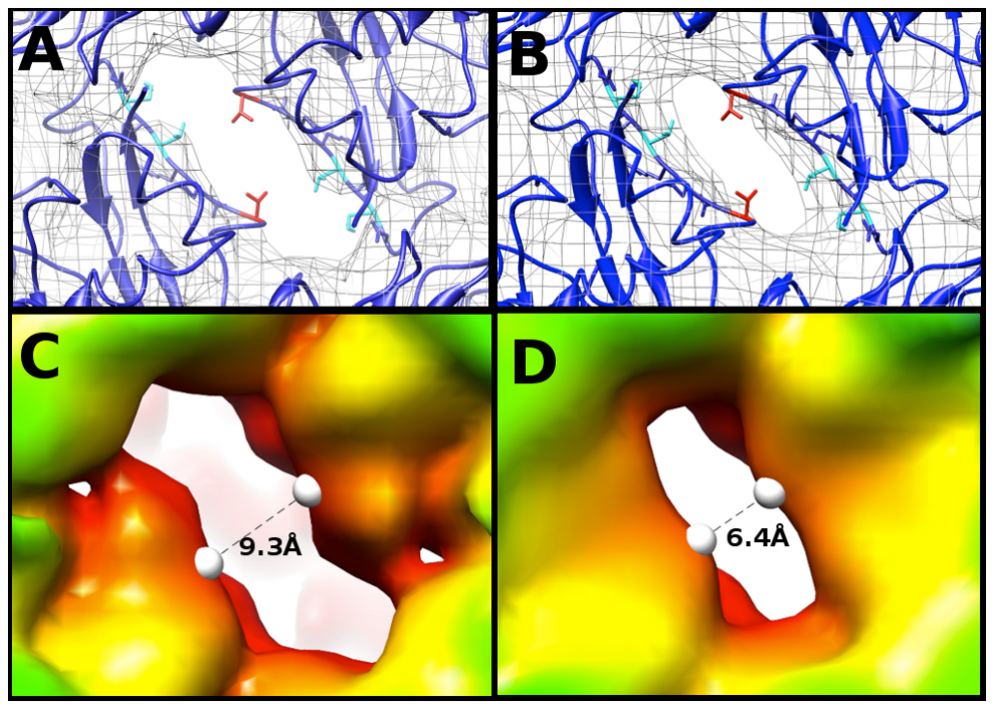
The two-fold axis of symmetry is wider in the A-particle than in the empty. (A,B) Fitting of the EV71 procapsid crystal structure (3VBU, dark blue) [30], into the 6.3 Å A-particle cryo-EM map (A) and empty capsid (B) (rendered in grey mesh at 1σ ). Val 58 of VP2 (shown in red) is outside the density in both fittings. Lys 52 and Thr 54 of VP2 (shown in cyan) are within the density of the empty capsid, but protrude from the density in the A-particle fit. Side chains in this region that are within the density of both fitted structures are shown in dark blue. (C) The 8.7 Å EV71 A-particle map has enlarged openings at the two-fold axis of symmetry that measure 9.9 Å in diameter, indicating that these channels are larger in the A-particle and not an artifact due to the higher quality of the 6.3 Å A-particle map. (D) The EV71 empty capsid has openings at the two-fold axis of symmetry 7.1 Å in diameter, consistent with the procapsid crystal structure.

To compare maps of similar resolution, the density map of the EV71 A-particle generated from ∼2000 particles was used to compare the diameter of the channel present at the two-fold axis with the same channel in the empty capsid. Fitting 3VBU into the A-particle revealed that two α-helices from adjacent VP2 proteins flank the opening of the two-fold channel, and a portion of the fitted structure (cc = 0.82) extends outside of the A-particle cryoEM density (Fig. 5A). Alternatively, the same crystal structure fitted into the empty capsid map (cc = 0.93) showed the α-helices fully within the density (Fig. 5B). Three residues of VP2 (Lys 52, Thr 54, and Val 58) are out of density in the fitted A-particle, whereas only Val 58 protrudes from the density in the fitting of the empty capsid (Fig. 5A,B). The two-fold opening in the A-particle has a measured diameter of 9.3 Å, whereas the empty capsid has an opening of only 6.4 Å, a 2.9 Å difference (Fig. 5C, D). This size difference suggests that the diameter of the channel is linked to the presence or absence of RNA.

### Calculating the Coulombic surface potential of the capsid

**Figure 6:**
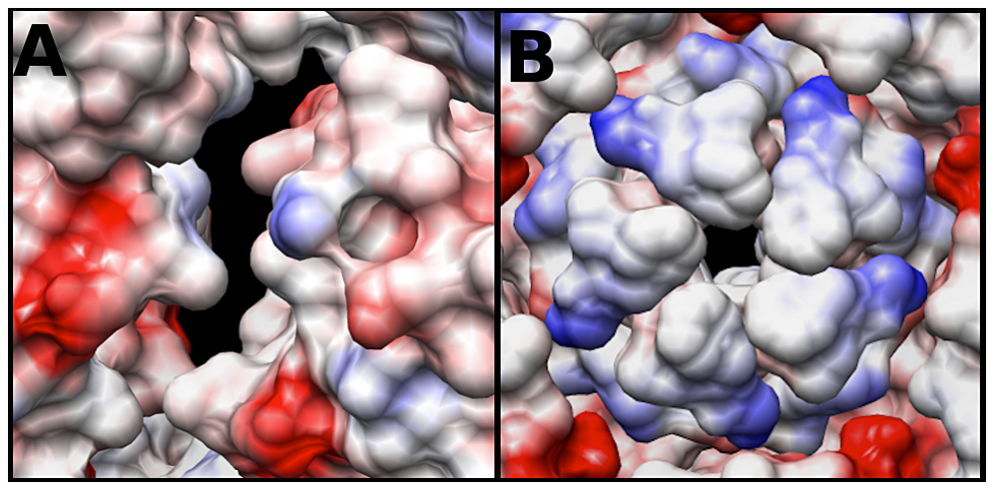
Coulombic surface coloring of the two-fold and five-fold axis. (A) The EV71 A-particle two-fold axis of symmetry has a high concentration of negatively charged residues (red) with minimal regions of positive charge (blue). This charge distribution will likely serve to aid in the egress of viral RNA by providing a “slippery” surface at the site of genome release. (B) The EV71 five-fold vertex is composed of neutral (white) and positive (blue) charges. In addition to the presence of a density plug at the base of the five-fold, these charges would repel the RNA suggesting the five-fold is not the site of genome expulsion.

The electrostatic potential of the fitted crystal structure (3VBU) was determine by Coulomb's Law, and the resultant surface charges were modeled on the fitted crystal structure. The A-particle shows that the interior surface has large patches of negative charge at the two-fold axis of symmetry with minimal interspersed positive charges (Fig. 6A). In contrast, the five-fold vertex is lined with positively and neutrally charged residues (Fig. 6B). These charge distributions are similar in the empty capsid (data not shown). The positive charges may attract the highly negative RNA towards the two-fold channel, whereas the large number of negatively charged amino acids would prevent the genome from becoming trapped in the channel. By this mechanism, the RNA may be positioned in the A-particle so that it can be released from a site between the two-fold and quasi three-fold axis (see discussion).

## Discussion

The new cryoEM structures presented here provide structural details of the EV71 lifecycle. A radial expansion takes place during uncoating to form the expanded A-particle and empty capsid. A previous study reported that heated mature EV71 particles, which had released their genome, crystallized isomorphously with procapsids [30]. The high correlation of the crystal structure to the cryoEM maps presented here, the nearly identical surface features of the A-particle compared to the empty capsid, and the lack of discernible difference density confirm the synonymous topographies of all types of expanded EV71 capsid forms.

However, the procapsid and empty capsid vary in protein composition. The 81 N-terminal residues of the uncleaved VP0 protein and the N-terminus of VP1 are located on the interior of the procapsid. In contrast, the downstream empty capsid that is formed after the cleavage of VP0 has extruded VP4 protein and externalized the N-terminus of VP1. These differences are reflected in the density at the interior surface of the empty capsid. In the procapsid crystal structure, 72 residues of the N-terminus of VP1 are disordered [30]. The cryoEM data and calculated difference maps show these residues constitute a significant portion of the pillar of density extending from the interior capsid surface towards the center of the capsid in both the A-particle and empty capsid. The remaining pillar density could be attributable to viral RNA. Volume calculations show that the disordered residues of VP1 can account for the volume differences shown in the calculated difference maps (Fig. 4C). Additionally, the procapsid has a flattened opening at the base of the canyon [30]. This opening was not observed in the A-particle or empty capsid map. The corresponding region was identified as difference density (Fig. 4C) suggesting that the N-terminus of VP1 exits the EV71 A-particle at the base of the canyon and remains externalized in the empty capsid.

Aside from the presence of viral RNA the most notable difference between the EV71 A-particle structure and the empty capsid, is the diameter of the two-fold channel. These differences may account for the lower correlation coefficient obtained from fitting the procapsid crystal structure into the A-particle (cc = 0.82) compared to the empty capsid fitting (cc = 0.93). The two-fold channel is formed by the separation of VP2 α-helices from adjacent protomers, during the transition of native virus to A-particle [30], [31]. Over all, these two-fold channels are broader and more open in the A-particle (Fig. 5), likely to facilitate the release of ordered RNA, an important feature as the termini of the picornavirus genome retains large amounts of secondary structure [40].

A conserved feature of picornaviruses is the amphipathic helix at the extreme N-terminus of VP1, which associates with host cell membranes as a critical event in RNA release [28], [41], [42]. In mature poliovirus, the N-terminus of VP1 is located near the five-fold axis [7]. However, in poliovirus A-particles the location of the N-terminus of VP1 shifts towards the tips of the three-fold propellers, and becomes externalized, as shown by antibody fragment labeling studies [43], [44]. The difference maps calculated here reveal two unfilled densities: one that is localized to the interior as a protein pillar, and another that is associated with the canyon (Fig. 4C). Volume calculations suggest that the N-terminus of VP1 is sufficient to fill these densities. The hole identified at the base of the canyon in the expanded EV71 procapsid is also present in the A-particle and empty capsid, and likely serves as the site for the externalization of the N-terminus of VP1 [30]. The location of the VP1 protein, its interaction with the viral genome, and the expansion of the two-fold channel would likely position the RNA to be released on a line between the two-fold axis and canyon. This site of genome egress has also been observed for poliovirus in a tomographic study [45]. The models presented here predict that concentrated patches of negatively charged amino acid residues, with interspersed regions of minimal positive charge, surround the two-fold channels. This local high concentration of negative charge has also been reported with human rhinovirus 2 [12]. These positive charges may serve to attract the viral genome, while the negative charges would prevent the RNA from sticking to the interior of the capsid, creating a “slippery” channel that begins at the two-fold axis and continues towards the base of the canyon for genome egress. Furthermore, the predicted positive “attractive” charges and the density plug below the five-fold vertex make the five-fold pore an unlikely site of genome release, as was once thought possible for other picornaviruses.

**Figure 7:**
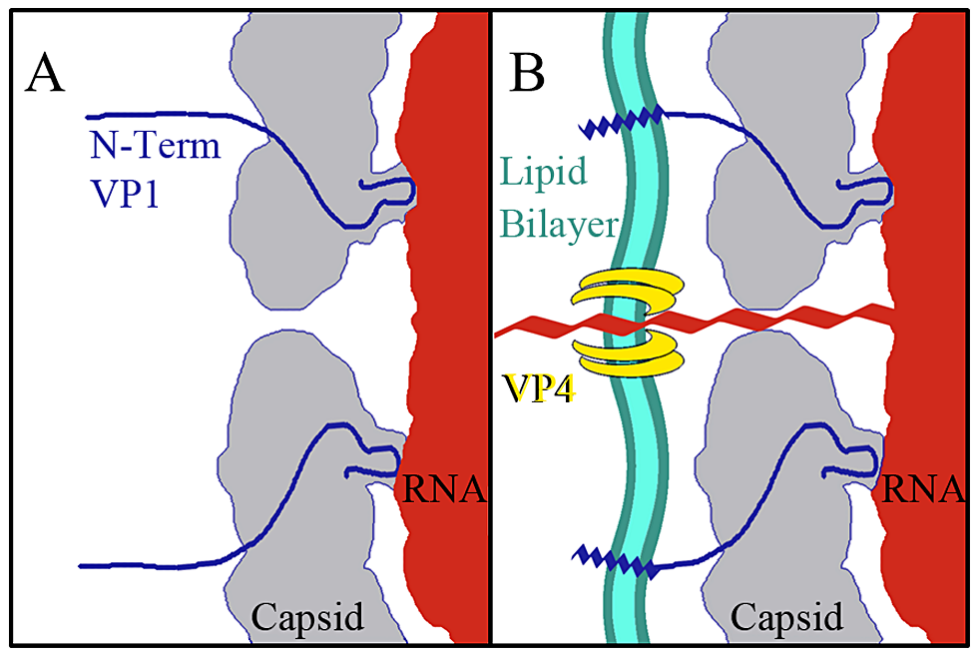
Model for EV71 uncoating. (A) Binding of a cellular receptor to the capsid of the mature virus (or heating) results in an expanded A-particle. This capsid conformation is characterized by the openings at each two-fold axis of symmetry, the externalization of the N-terminus of the VP1 protein (residues 1–72) from the base of the canyon, and the expulsion of the VP4 protein. (B) If the A-particle forms in proximity to a cellular membrane the VP4 proteins form a channel through which the RNA can enter the host cell cytoplasm. The exposed N-terminii of VP1 proteins anchor the A-particle to the membrane via amphipathic helices.

The data presented here provide for a more complete model of picornavirus uncoating. In the mature EV71 capsid the two-fold channels are closed. The virus remains in a metastable state, transiently exposing the N-terminus of VP1 in a process deemed “breathing”, and is capable of withstanding the harsh extracellular environment [46], [47]. When the capsid interacts with its receptor on a host cell membrane the pocket factor is expelled from the base of the canyon [26]. This release allows the capsid to expand and open the two-fold channels, leading to formation of the A-particle, in which a portion of the N-terminus of VP1 is tethered to the RNA genome. The VP4 proteins are expelled, and insert into the membrane to form a pore (Fig. 7). The extreme N-termini of VP1 extend from the opening at the canyon base, anchoring the amphipathic helices into the membrane. These events poise the RNA for release. It has been proposed that at one unique site on the A-particle the thin protein layer between the openings at the two-fold axis and canyon base breaks, creating a large pore for genome release [30]. The structures presented here indicate that the movement of the N-terminus of VP1 away from the stabilizing VP2 α-helices at the two-fold, and towards the base of the canyon regulates the formation of a gateway in the protein shell, through which the genome is released.

## Materials and Methods

### Virus propagation and purification

Confluent HeLa cell monolayers were infected with EV71 strain 1075/Shiga at an MOI of 0.1 in culture with DMEM supplemented with 2.5% fetal bovine serum. Cells and media were harvested 24 hours post infection and subjected to three freeze-thaw cycles. To remove cell debris, the lysate was centrifuged at 13K rpm in a SLA1500 rotor at 4°C for 15 minutes. After the addition of 8% PEG 8K and 0.5 M NaCl virus was precipitated overnight at 4°C and then centrifuged in a SLA1500 rotor (4°C, 13K rpm, 45 minutes). Pellets were resuspended in purification buffer (10 mM Tris-HCl, 200 mM NaCl, 50 mM MgCl, pH = 7.5) and 0.05 mg/ml DNase was added. The suspension was incubated at room temperature for 10 minutes with gentle rocking. After incubation 0.1 M EDTA pH = 8.0 was added (10% total volume), the pH was readjusted using ammonium hydroxide, and supernatant was cleared by low speed centrifugation. The supernatant was then pelleted through a 30% sucrose-buffer cushion (50.2ti rotor, 48K rpm, 4°C, 90 minutes). The pellet was resuspended in 2 mL purification buffer, centrifuged at 4000K to remove any remaining cellular debris, and applied to a 10–35% potassium tartrate step gradient for final purification by ultracentrifugation (36K rpm, 4°C, 2 hours, SW41 rotor). Two distinct bands of virus were collected by side puncture and buffer exchanged into purification buffer. As previously reported [22], [30] the upper band consisted of procapsid, characterized by the presence of uncleaved VP0 and lack of genomic material. The lower band consisted of native virus comprised of VP2 and VP4, with packaged genome. Each species was diluted to a concentration of 0.1 mg/ml for storage at 4°C in purification buffer.

### Cryo-electron microscopy

To produce A-particles and empty capsids, purified mature EV71 particles concentrated to 0.1 mg/mL in 10 mM Tris-HCl, 20 mM NaCl, 5 mM MgCl, pH = 7.5 buffer were heated to 56°C for 12 minutes. The presence of both A-particles and empty capsids was confirmed by negative stain electron microscopy using the JEOL 1400 transmission electron microscope in the microscopy imaging shared core facility at the Pennsylvania State University College of Medicine. Aliquots of sample were blotted onto glow discharged holey carbon Quantifoil electron microscopy grids and plunged into a mixture of liquid ethane and propane (50∶50) cooled by liquid nitrogen using a FEI Vitrobot mark III freezing robot (FEI, Hillsboro, OR) [48]. Data were collected in a FEI Tecnai TF-20 FEG electron microscope operating at 200 kV with magnification 50,000×, on Kodak SO-163 film (Kodak, Rochester, NY). The films were scanned using a NIKON Super Coolscan 9000 with a sampling rate of 6.35 micron/pixel to generate a pixel size of 1.27 Å at the sample. Empty capsids were selected individually, whereas A-particles were selected using semi-automated boxing with the e2poxer.py module of EMAN2 [49]. The data were then linearized, normalized, and apodized using AUTO3DEM [50]. During the reconstruction process using AUTO3DEM, phases were flipped to correct for contrast transfer function (CTF). The resolution of each map was assessed by comparing half-dataset maps using Fourier shell correlation (FSC) at a cutoff of 0.5 (Fig. S1). The cryo-EM maps of the A-particle and empty capsid have been deposited in the Electron Microscopy Data Bank (accession codes EMD-5465 and EMD-5466, respectively). A similar reconstruction procedure was used to calculate a second A-particle map from 2000 particles that was limited to 8.7 Å resolution. This map was used for comparisons with the empty capsid map (Table 1).

### Assessment of the absolute pixel size of density maps

The asymmetric unit of the expanded naturally occurring EV71 empty capsid (3VBU) was obtained from the PDB and used to generate an icosahedral map in Chimera with a calculated pixel size = 1.3 Å [25]. The pixel size of the empty capsid cryoEM density map (σ = 1.00) was altered by 0.01 increments while assessing the correlation coefficient (cc) compared to the calculated map with a finite pixel size = 1.3 Å. The value that corresponded to a cc closest to 1 provided the absolute pixel size of both the A-particle and empty capsid density maps (pizel size = 1.27 Å, Table 2).

**Table 2:** Assessment of the absolute pixel size of the CryoEM maps.

Pixel Size (Å)	Correlation Coefficient
1.20	0.6935
1.25	0.9306
1.26	0.9437
1.27	0.9491
1.28	0.9481
1.30	0.9280
1.40	0.6490

### Fitting crystal structure into density maps

To obtain an accurate fit for the A-particle, a single asymmetric unit of the EV71 naturally occurring empty capsid (3VBU) was preliminarily fitted into the A-particle density map (σ = 1.00) using “Fit to Map” in Chimera and icosahedral symmetry operators were applied to generate the structure of the entire capsid. The resulting full map was then docked as a rigid body into the A-particle cryoEM density using Situs to obtain a refined fit [33]. This process was repeated for the density map of the empty capsid. The coordinates of the protomers fitted into the empty capsid and A-particle were deposited in the Protein Data Bank (accession codes 3J23 and 3J22, respectively).

### Density difference maps, surface charge analysis, and volume measurements

A difference map was calculated by subtracting the density of the 9 Å EV71 empty capsid (pixel size = 1.27 Å) from the density of the EV71 A-particle map of similar quality [51]. A calculated procapsid map was produced using Situs by mass-weighting the atoms and writing the map to a resolution of 6.5 Å. This map was subtracted from the density of the A-particle and the empty capsid to produce two separate difference maps in Situs [51]. Volume measurements of the disordered N-terminus of VP1 and the empty capsid minus procapsid difference density map were completed using the “Measure Volume and Area” tool in Chimera [52]. Chimera was used to predict the Coulombic charges of the fitted crystal structure. The capsid-RNA interactions in the A-particle, measurements, and images were completed using Chimera with maps displayed at a contour level of 1 sigma [52].

## Supporting Information

Figure S1 **Heating native EV71 particles produced a mixture of A-Particles and empty capsids.** (A) A mixed population of EV71 A-particles and empty capsids negatively stained with uranyl formate (UF) at 20,000×. (B) Cryo-electron micrograph (50,000× magnification) of vitrified EV71 particles after heat treatment with both A-particles and empty capsids present. The A-particles appear to contain a consistent amount of genetic material, whereas the empty capsids are fully devoid of nucleic acid.
(TIF)

Figure S2 **Resolution determination by Fourier shell correlation.** The resolution of the density maps of the EV71 (A) A-particle and (B) empty capsid were assessed at a Fourier shell correlation cutoff of 0.5. The A-particle reached 6.3 Å resolution and the empty capsid reached a resolution of 9.2 Å.
(TIF)
