# Correction: The Enterovirus 71 A-particle Forms a Gateway to Allow Genome Release: A CryoEM Study of Picornavirus Uncoating

**DOI:** 10.1371/annotation/e92d19e0-996a-4bfa-afdd-20dce770ed75

**Published:** 2013-09-19

**Authors:** Kristin L. Shingler, Jennifer L. Yoder, Michael S. Carnegie, Robert E. Ashley, Alexander M. Makhov, James F. Conway, Susan Hafenstein

The Materials and Methods section of the manuscript incorrectly refers to a virus strain named 1075/Shiga. The correct virus strain name is 1095/Shiga. 

