# Supplementary figures and images for: The Enterovirus 71 A-particle Forms a Gateway to Allow Genome Release: A CryoEM Study of Picornavirus Uncoating
Source: PLoS Pathog. 2013 Mar 21;9(3):e1003240. doi: 10.1371/journal.ppat.1003240 (PMC3605244; doi:10.1371/journal.ppat.1003240)

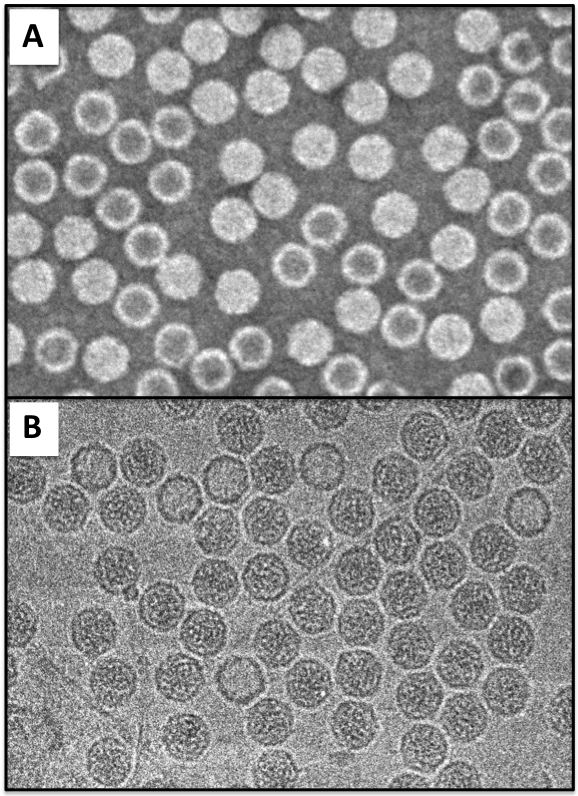

Supplement: Figure S1 — Heating native EV71 particles produced a mixture of A-Particles and empty capsids. (A) A mixed population of EV71 A-particles and empty capsids negatively stained with uranyl formate (UF) at 20,000×. (B) Cryo-electron micrograph (50,000× magnification) of vitrified EV71 particles after heat treatment with both A-particles and empty capsids present. The A-particles appear to contain a consistent amount of genetic material, whereas the empty capsids are fully devoid of nucleic acid. (TIF) [file ppat.1003240.s001.tif]

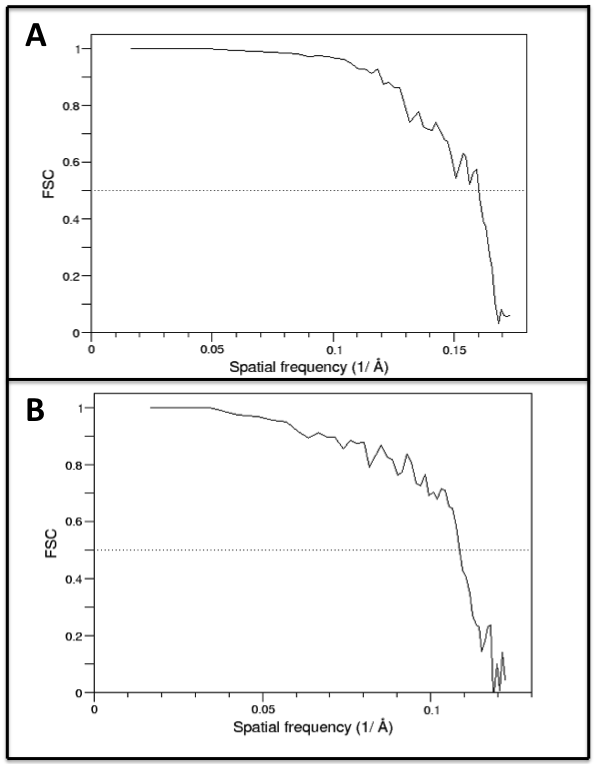

Supplement: Figure S2 — Resolution determination by Fourier shell correlation. The resolution of the density maps of the EV71 (A) A-particle and (B) empty capsid were assessed at a Fourier shell correlation cutoff of 0.5. The A-particle reached 6.3 Å resolution and the empty capsid reached a resolution of 9.2 Å. (TIF) [file ppat.1003240.s002.tif]
